# The Struggle for a Living

**Published:** 1883-06

**Authors:** 


### The Struggle for a Living.

The following is taken from the Presidential address to the Maine Medical Association by the late Wm. Warren Greene, M. D. (*N. Y. Med. Record*, March 24, 1883.)

"Witness the large number of doctors in every city struggling for mere existence, and see how few out of the whole number really do the work. See how in almost every country village a full practice for one or two good men is piece-mealed by sharp and often acrimonious competition, to the detriment of all.

"It would seem that in a calling so high, so noble, so sacred, men fit for such ministry should be sought for; but the great question of the young graduate is not 'Who wants me?' but 'Who will employ me?' not 'Who needs me?' but 'Where can I get a living?' In the case of four physicians dying, each in a country village, during the last year, I am credibly informed that in one instance two, in another three, in the third five, and in the fourth case seven new men came to look the field over within ten days after the doctor's death--sometimes before the burial. In one case ten attended the funeral, and in another the widow had three letters from aspirants for the vacant place while the dead body of her husband still lay in the house.

"It is a hackneyed saying, with which too many ears are tickled, that 'there is always room for good men.' Applied to the present condition of our profession, it is false. Were only good men and the best men admitted, it would undoubtedly be true. But all over the land, in city and country, are well educated, cultured gentlemen, honest and loyal, striving in vain to secure a competence—yes, a bare living even—and too often is disappointment mingled with shame and mortification at the success of ignorant and unprincipled rivals. I have said that the evil results of this excess in numbers are manifold. It leads to over-practice and bad practice. The man who is hard pushed, who has few patients, and needs more, is tempted to make much of little; to magnify the importance of his cases, both in his own mind and to his patrons; to make uncalled-for visits, and to give too much medicine; and unnecessary medication soon ceases to be rational. Patients are injured in mind and body. The community is injured by teaching the people to attach undue importance to trivial diseases, and to over-estimate the value of treatment therein. Legitimate, honest practice suffers in reputation; money is obtained under false pretences."

